# Serum micro-RNA Identifies Early Stage Colorectal Cancer in a Multi-Ethnic Population

**DOI:** 10.31557/APJCP.2020.21.10.3019

**Published:** 2020-10

**Authors:** Jessica Shiosaki, Maarit Tiirikainen, Karolina Peplowska, David Shaeffer, Michio Machida, Kazuhiro Sakamoto, Makoto Takahashi, Kuniaki Kojima, Junji Machi, Peter Bryant-Greenwood, Scott K Kuwada

**Affiliations:** 1 *John A. Burns School of Medicine, University of Hawaii, 651 Ilalo Street, MEB, Honolulu, Hawaii, United States. *; 2 *University of Hawaii Cancer Center, 01 Ilalo Street, Honolulu, Hawaii, United States. *; 3 *Juntendo University, 3-1-3 Hongo, Bunkyo-ku, Tokyo, Japan. *; 4 *National Jewish Health, 1400 Jackson Street, Denver, Colorado, United States.*

**Keywords:** Colorectal cancer, micro-RNA, diagnosis, multi-ethnic

## Abstract

**Objective:**

Certain microRNAs (miR) have been previously described to be dysregulated in cancers and can be detected in blood samples. Studies examining the utility of miRs for colon cancer screening have primarily been performed in ethnically homogeneous groups of patients, thus the performance of miRs in multiethnic populations is unknown.

**Methods:**

Four miRs were selected that were shown to be aberrantly expressed in the blood or stool of patients with colorectal cancer (CRC) of various ethnicities. In this study, the ability of these miRs to discern early stage CRC was determined in a previously untested multiethnic population of 73 CRC cases and 18 controls.

**Results:**

The ratios of non-vesicular to extracellular vesicular levels of miR’s -21, -29a, and -92a were statistically and quantitatively related to CRC stage compared to controls.

**Conclusion:**

Serum levels of miR-21, miR-29a and miR-92a were able to significantly detect early stage CRC in a multiethnic and previously untested population.

## Introduction

Colorectal cancer (CRC) is a cause of significant morbidity and mortality in developed countries where the incidence is highest. Multiple screening modalities are available, but compliance with these programs remains modest. In the U.S., only about two-thirds of eligible patients are up to date with CRC screening (Smith et al., 2007). Colonoscopy, considered the gold-standard screening test for CRC, offers at least a 70% reduction in CRC (Winawer et al., 1993), but is invasive and expensive making universal acceptance and accessibility challenging. Barium enema and computed tomographic colonography lack data demonstrating a reduction in mortality. Fecal occult blood testing reduces the incidence of colorectal cancer but suffers from poor sensitivity for CRC detection (Mandel et al., 1993). Fecal DNA testing and immunochemical detection of hemoglobin offer improved sensitivity for CRC than heme oxidase-based stool tests but have yet to demonstrate a reduction in CRC mortality. CEA, a protein marker in the blood, is currently used for monitoring CRC patient therapy, but is not recommended for screening as it is rarely elevated in early stages of CRC and cannot differentiate between benign and malignant polyps (Lech et al., 2016). The majority of CRC is discovered after it has spread to other parts of the body or has grown large enough to become symptomatic, both of which diminish the potential for cure. On the other hand, early-stage CRC (Stage I and II) can be cured with surgical resection alone with an overall 5-year survival rate of 90%. Thus, an effective screening strategy capable of detecting asymptomatic and early stage CRC that has high acceptance and availability remains elusive. 

Circulating biomarkers in the blood could offer a simple, less invasive and widely accessible first-line screening tool for CRC. Such a test could identify individuals who are at highest risk of harboring a colorectal neoplasm, and would then be referred for colonoscopy. 

One such potential biomarker is miR, short non-coding segments of RNA that regulate cell development, differentiation, proliferation, apoptosis and metabolism through post-transcriptional gene regulation. miRs have been implicated in cancer progression, contributing to tumorigenesis, angiogenesis, and metastasis through key signaling pathways such as Wnt/β-catenin, EGFR, TP53, and TGF-β. (Calin et al., 2005; Volinia et al., 2006; He et al., 2007; Schetter et al., 2008). These effects have been hypothesized to occur via exosomes transferring miR between cells (Calin et al., 2005; Volinia et al., 2006; He et al., 2007; Schetter et al., 2008). A number of CRC studies showed that aberrant tissue levels of miRs, compared with normal tissue, correlated with prognosis, disease progression, and therapeutic outcome (Schepeler et al., 2008; Schetter et al., 2008; Schetter et al., 2009; Karaayvaz et al., 2011; Kjaer-Frifeldt et al., 2012; Weissmann-Brenner et al., 2012; Tsai et al., 2013; Zhang et al., 2013; Christensen et al., 2014). Important for screening applications, circulating miRs have been detected in the plasma and serum of CRC patients (Chen et al., 2008; Mitchell et al., 2008; Ng et al., 2009). The stability of cell-free miRs in body fluids lends readily to their potential as biomarkers for noninvasive diagnosis of cancer. 

The incidence of CRC is climbing in Asian countries with some rivaling the incidence in Western developed countries. The studies of miR as biomarkers for CRC detection have been performed in fairly ethnically homogeneous study populations making translation of results across various ethnic groups problematic. We thus decided to test select miRs, shown to be aberrantly expressed in the blood of CRC cases across various ethnicities, in a previously untested ethnically diverse group of CRC cases.

Based on previous studies in various but relatively homogeneous ethnic Asian populations, miR’s -21, -29a, -92a, and -221 were commonly overexpressed in CRC (Chen et al., 2008; Uratani et al., 2016). These four miRs have been implicated in colorectal tumorigenesis as well. Investigations of miR -21 demonstrated its role in tumor growth and metastases through downregulation of PDCD4 which promotes cell migration and angiogenesis by increasing VEGF levels (Krichevsky and Gabriely, 2009). Additionally, elevations of miR -21 in cancerous tissue and serum portends poorer overall survival (Toiyama et al., 2013). miR -92a and -221 enhance migration and metastasis of cancer cells (Bell and Taylor, 2017). miR -92a-3p has been shown to promote CRC metastasis by targeting the CDH1 gene (Lin et al., 2013). Studies have shown involvement of these miRs in key cancer signal transduction pathways: miR-221 in the KRAS pathway, miR -92a in EGFR activation and miR -21 in TGF-β signaling (Sebio et al., 2013; Shirafkan et al., 2018). 

## Materials and Methods

*Study Design*

IRB approval for specimen collection was obtained at four sites: Kaiser Moanalua Medical Center in Honolulu, HI; The Queen’s Medical Center (QMC) in Honolulu, HI; Juntendo University in Tokyo, Japan; and Juntendo University in Nerima, Japan. The two enrollment sites in Hawaii serve multi-ethnic patient populations that reflect Hawaii’s multi-ethnic distribution (10.2% Native Hawaiian or Pacific Islander alone, 25.6% white, 37.6% Asian alone, 2.2% Black or African American (Census, 2020)). Visits to Juntendo University, as well as Kaiser Moanalua, were performed to ensure adherence to quality control standards there. 

Enrollment began in 2007, and enrollment targets were met in December 2010. Eligibility criteria for this study include: 18 to 89 years of age; not pregnant; not incarcerated; no history of inflammatory bowel disease; no prior history of CRC; no history of any type of cancer in the last 2 years; and no history of carcinoid tumors, mesenchymal tumors, colitis, or diverticulitis. Eligible individuals with or without CRC were approached by a member of the research team, the research was fully explained, and individuals were invited to participate. If they agreed to participate, they were asked to read and sign an informed consent. After consent was obtained, a venipuncture was performed by a certified lab technician, and 10 milliliters of blood was drawn. The blood was allowed to clot for 10 minutes, and then the tube was centrifuged for 10 minutes at 1,300 g. The serum was drawn off and frozen at -80^o^C. This process was always completed within 1 hour of collection. Specimen containers were labeled with only a study number for de-identification purposes. The specimen collection process occurred prior to any definitive surgical or endoscopic procedure to ensure the presence of circulating cancer-associated miR. If, after the definitive procedure, the individual was found not to fit into the expected group, this individual’s specimen was excluded from the research. 

Relevant clinical and pathological information about the enrolled participants were ascertained from operative notes and pathology reports. Patients in the endoscopy normal group were further categorized based on the presence or absence of polyps. Information stored in the electronic database includes: study number, patient age, pertinent medical history, findings at the time of the procedure, tumor location if present, histologic diagnosis, and complete pathological staging. 

*Vesicle Extraction and miR Purification Procedures*

Select miRs, miR -21, -221, -29a and -92a, which were previously validated in other multi-ethnic clinical studies (Ng et al., 2009; Huang et al., 2010b; Pu et al., 2010a; Ahmed et al., 2012; Luo et al., 2013) (Huang et al., 2010a) (Liu et al., 2013) (Pu et al., 2010b), were measured in both the circulating extracellular vesicles (EV) and non-vesicular supernatant from 91 total serum samples. Sera were collected from 18 healthy control patients and 73 CRC cases (20 stage I, 20 stage II, 18 stage III, and 15 stage IV CRC). The EV/non-vesicular fractionation and the extraction of total RNA containing miRs were performed using the exoRNAeasy Serum/ Plasma kit (Qiagen) per manufacturer’s instructions. This kit enables isolation of a mixed population of blood microvesicles, including exosomes. We therefore use the terminology “extracellular vesicles” (EV) to define the source of miRs in this study. Serum miRs were quantified in triplicate using pre-designed TaqMan miR assays (Applied Biosystems) and quantitative real time PCR analysis. The miRs of interest were reverse transcribed to cDNA using the TaqMan MicroRNA Reverse Transcription Kit (Applied Biosystems) and preamplified using the TaqMan PreAmp kit per manufacturer’s instructions. Before extraction, the sera were spiked with *C. elegans* mir-39 to allow for monitoring of the expression efficiency and miR recovery between the samples. Water-only controls containing all reagents but no cDNA were used for the quantitative real time PCR reactions.

*Statistical Analyses*

Expression levels of the four miRs were normalized using an exogenous spiked-in miRNA (C. elegans mir-39). Results were expressed as means with SEM. Two-tailed Mann-Whitney analyses were used to compare the differences between serum miR expression. Non-vesicular to EV ratios were calculated and analyzed to detect differences between healthy controls and patients with stages I-IV CRC. Area under the curves (receiver-operator curces) were reported with 95% confidence intervals.

## Results

We examined of previously identified miRs (miR-21, miR-221, miR-29a, and miR-92a) that were aberrantly expressed in CRC compared with controls (Ng et al., 2009; Huang et al., 2010b; Pu et al., 2010a; Luo et al., 2013; Ahmed et al., 2012; Huang et al., 2010a; Liu et al., 2013; Pu et al., 2010b). These discovery studies utilized a strategy in which CRC tissue specimens were first used to find miRs that were aberrantly expressed compared with normal colon tissue, and then to see which of these miRs was aberrantly expressed in the blood from the same patients. Validation of such studies in previously untested CRC cases and controls are required to determine the utility of these miRs in CRC screening and detection. Thus, we examined these miRs in our previously untested and multiethnic population of CRC cases. Particular attention was paid in determining the utility of these miRs as markers of early stage CRC. To this end, serum samples were collected from 73 CRC cases, which included 40 stage I-II CRC cases, and 18 control patients (no evidence of CRC or advanced colorectal polyps on first colonoscopy) (Table 1).

**Figure 1:**
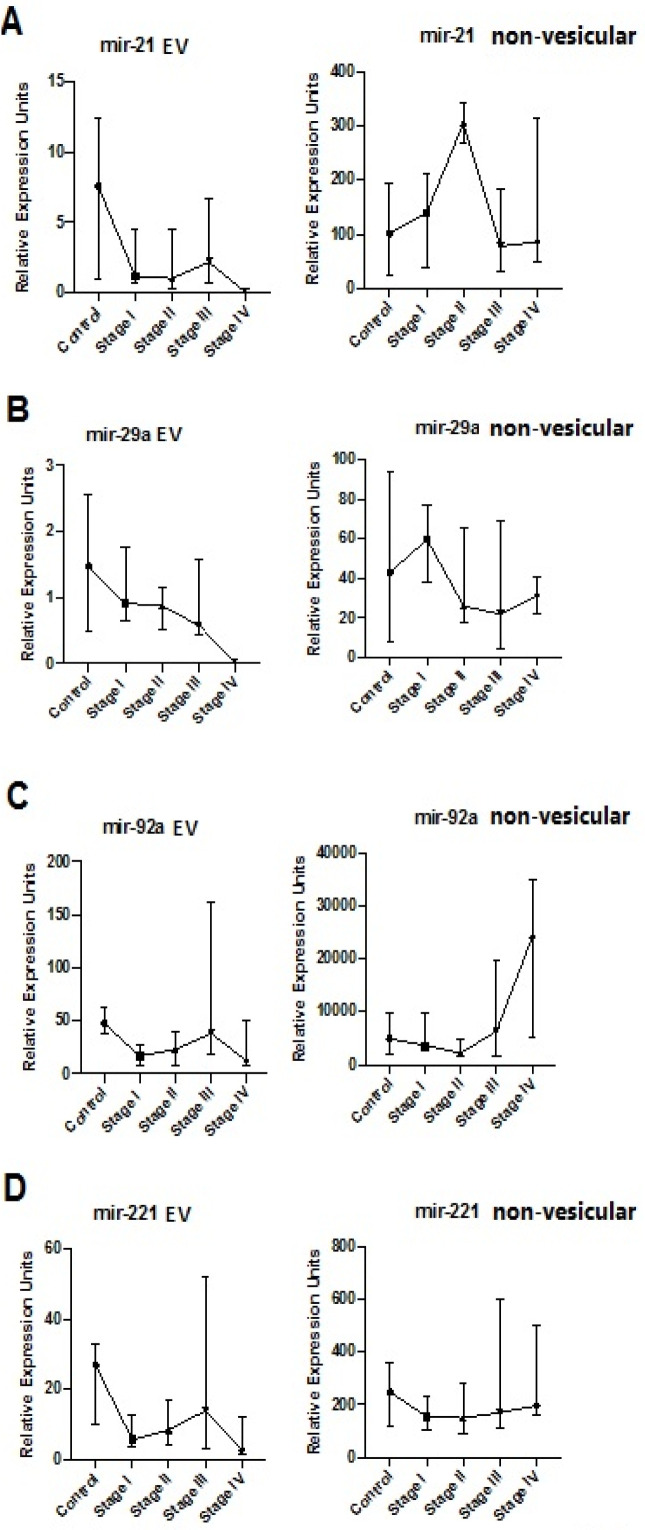
Extracellular Vesicular and Non-Vesicular Expression of Selected miRs in Serum Samples of Colorectal Adenocarcinoma Patients by Stage. The graphs show the extracellular-vesicular (vesicular) and non-vesicular expression of miR -21 (A), -29a (B), -92a (C), and -221 (D) in serum samples for CRC cases and controls. The data are represented as medians with 95% confidence intervals

It is important to note that the miRs of interest were extracted and purified from extracellular vesicles and miRs have been found in a variety of extracellular vesicles included exosomes, microvesicles and apoptotic bodies (Than et al., 2018). Using serum samples normalized for spiked-in C. elegans mir-39, non-vesicular and EV expression levels of miR-21, miR-221, miR-29a, and miR-92a were determined for various stages of CRC and controls (Figure 1A-D). Non-vesicular levels of miR-21, miR-221, and miR-29a for CRC stages I-IV remained relatively similar to controls (Figure 1A-D). However, non-vesicular levels of miR-92a showed a trend for increasing levels for stage III CRC and reaching significance for stage IV CRC compared with controls (Figure 1C). In general, there was a decline in EV levels of all four miR’s with progression of CRC stage, especially for stage IV, compared with controls (Figure 1A-D). 

**Figure 2:**
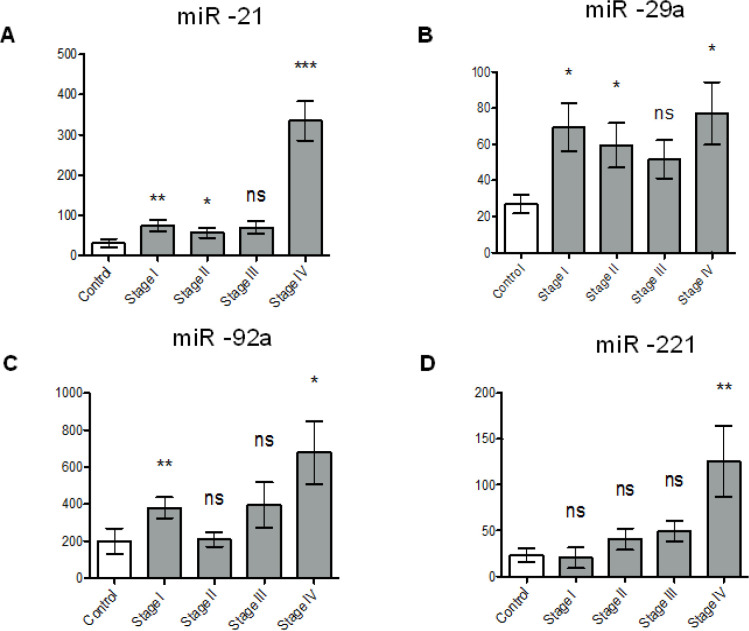
Ratio of Non-Vesicular to Extracellular Vesicular Expression Levels of Selected miRs in Serum Samples of CRC Cases by Stage. The graphs show the non-vesicular to EV expression ratios in miR -21 (A), -29a (B), -92a (C), and -221 (D). Data is represented as means with standard error of the means

**Figure 3:**
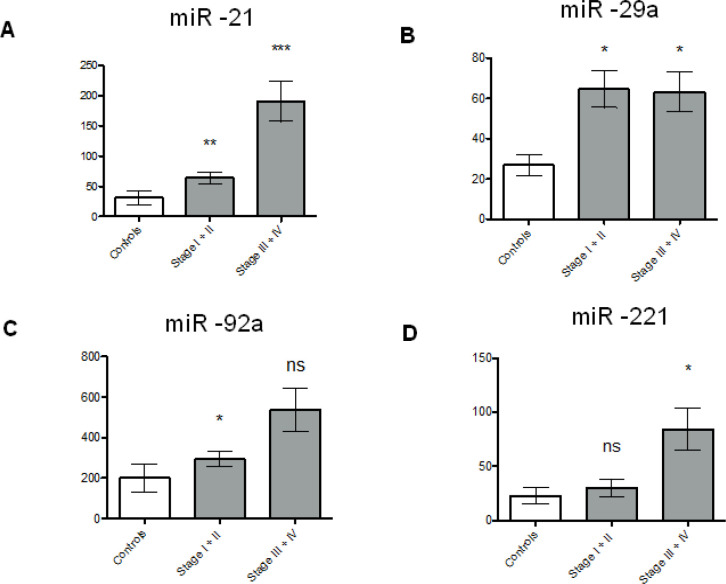
miR -21, -29a, -92a, -221 Expression Levels in Early and Late Stage CRC Compared to Controls. The graphs show the non-vesicular to EV expression ratios in miR -21 (A), -29a (B), -92a (C), and -221 (D) for controls versus early stage (I-II) and late stage (III-IV) CRC cases. Data are represented as means with standard errors of the means

The differing trends in EV and non-vesicular levels for miR-21, miR-29a, miR-221 and miR-92a by CRC stage suggested potentially dynamic relationships between the EV and non-vesicular compartments for each of the miRs, which to our knowledge has not been explored before. Thus, the ratios of the non-vesicular to EV levels for the four miRs was examined as a function of CRC stage. miR-21 and miR-29a demonstrated significantly higher non-vesicular to EV ratios in stages I, II and IV compared to controls (Figure 2 A and B). miR-92a ratio expression levels were higher in stage I and IV compared to controls, and miR-221 expression was only significantly higher in stage IV compared to controls (Figure 2 C and D). Significantly higher levels of miR-21, miR-29a and miR-92a were detected in earlier stages (I-II) of colorectal cancers and of miR-21, miR-29a, and miR-221 were detected in later stages (III-IV) compared to controls (Figure 3 A-D). 

**Figure 4:**
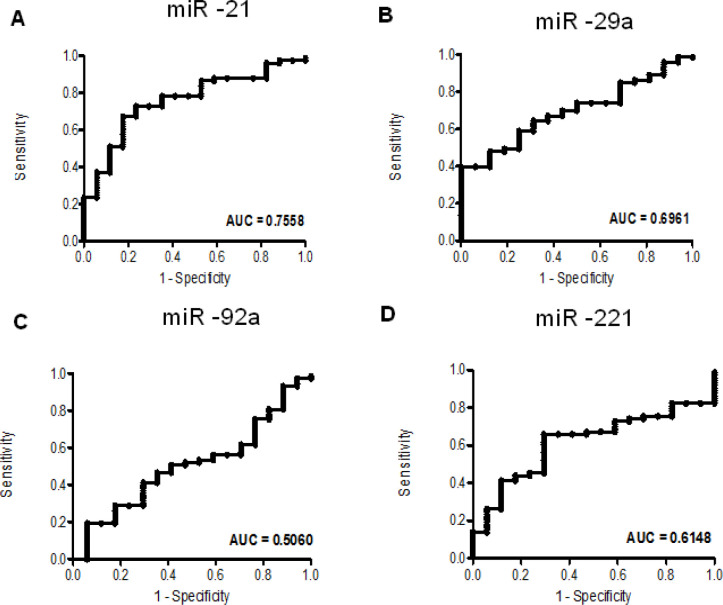
Receiver Operator Characteristic (ROC) Analyses. The graphs show receiver operator characteristic curves for miR -21 (A), -29a (B), -92a (C), and -221 (D) in differentiating between healthy controls and CRC (all stages)

To examine the potential utility for these serum miRs to detect CRC, we performed receiving operator characteristic (ROC) analyses. ROC analyses indicated the highest utility for miR-21 alone to distinguish between healthy patients and all stages of CRC patients, with an AUC of 0.7558 (95%CI = 0.6388 to 0.8728) (Figure 4). Specifically for the detection of early stage CRC, miR-21 had an AUC of 0.7265 (95%CI = 0.5785 to 0.8744). Using the ROC to determine the cut-off point, miR-21 performed with a 72.6% sensitivity and 70.6% specificity. A combination of miR -21, -29a, and -92a for early stage CRC detection yielded an AUC of 0.6385 (CI = 0.5474 to 0.7295). 

**Table 1:** Study Population Characteristics

Characteristic	CRC (n=73)	Controls (n=18)
Asian (%)	83	70
Native-Hawaiian/Pacific Islander (%)	4	15
White (%)	12	15
African American (%)	1	0
Average Age at Diagnosis (years)	65.8	62
Gender (%)	F = 60; M = 40	F = 42; M = 58
Site (%)	Right = 33	
	Left = 67	
	Rectum = 17	
Stage I	N = 20	
Stage II	N = 20	
Stage III	N = 18	
Stage IV	N = 15	

## Discussion

Several studies used profiling of CRC and control patients and showed differential expression of circulating miRs, such as miR-29a, miR-92a and miR-221, and therefore of potential use as non-invasive biomarkers for CRC screening (Ng et al., 2009; Huang et al., 2010b; Pu et al., 2010a). 

Only a few studies to date have validated miR detection in untested CRC cases: One study of 12 miRs that were differentially expressed in patients with CRC versus controls (miR-29a, -106b, -133a, -342-3p,-532-3p, -18a, -20a, -21, -92a, -143, -145, -181b) was performed in patients with CRC (n = 80), advanced colon polyps (n = 50) and controls (n = 144) (Luo et al., 2013). Nine of the twelve miRs (miR-18a, -20a, -21, -29a, -92a, -106b, -133a, -143, -145) were found to be differentially expressed in CRC patients and controls in the validation samples. The optimism-corrected area under the curve was 0.745 (95% confidence interval: 0.708–0.846). Unfortunately, none of the selected miRs showed significant differential expression between advanced adenoma patients and healthy controls. Another study examined the performance of 9 miRs overexpressed (miR -7, -17-3p, -20a, -21, -92a, -96, -183 -196a, -214) and 6 miRNA’s underexpressed (miR – 124, -127-3p, -138, -143, -146a, -222) in CRC’s versus controls (Ahmed et al., 2012). Using the 15 selected miRs a sensitivity of 90% and specificity of 95% was achieved for CRC cases versus controls (Ahmed et al., 2012). These two aforementioned studies were performed in predominantly white patients.

Previous studies used a similar approach in Chinese patients which provides some insight into the performance of published CRC-associated miRs in Asian populations (Huang et al., 2010a; Pu et al., 2010b; Liu et al., 2013). Identifying signatures of ethnicity-specific miRs may improve their reliability and validity when used as a screening test in ethnically diverse populations. miR polymorphisms have in fact revealed differences in miR sequences amongst different ethnicities that affect differential expression and function (Ye et al., 2008). Perhaps most illustrative of this point was a pilot study by Zhao et al., (2010) in which specific plasma miRs were shown to discriminate between breast cancer patients and healthy controls, but with significant differences in the performance of these miRs between African-American and white patients. In a Chinese study, Huang et al., (2010b) found plasma levels of both miR -29a and miR -92a to have diagnostic value for advanced CRC neoplasia with an area under the curve (AUC) value of 83% using receive-operator analysis. Furthermore, the miR -29a and miR -92a combination yielded a 73% sensitivity and 79.7% specificity in distinguishing advanced adenoma patients from controls. Uratani et al., (2016) demonstrated serum miR’s -21, -29a and -92a to have sensitivities of 73%, 71%, and 65%, respectively, for colonic adenoma patients vs. healthy controls, suggesting the potential utility for these three miRs for the early detection of high-risk polyps and CRC. Pu et al., (2010a) observed the correlation between mir-221 levels as a biomarker to identify CRC patients as well as a prognostic factor for poor survival. Specifically regarding early stage colon cancer, a recent study in China found exosomal miR-125a-3p to have a higher expression compared to healthy controls, with an AUC of 0.855 when combined with CEA (Wang et al., 2017). These studies provided our rationale for determining if serum levels of miR-21, miR-221, miR-29a, and miR-92a could identify early stage CRC patients in an ethnically diverse, previously untested and predominantly Asian population. Previous studies of the miRs we selected, miR-21, miR-29a, miR-92a, and miR-221, have not to our knowledge been validated in an untested and multiethnic study population. 

While our study did not find consistent discrimination between EV or non-vesicular serum levels of miR-21, miR-29a, miR-92a and miR-221by stage of CRC, we found that the ratios of the levels of non-vesicular to EV levels of miR’s -21, -29a, and -92a, but not miR-221, were significantly higher for early stage (I-II) CRC’s versus controls. The possible underlying explanations for our results showing increasing ratios of serum non-vesicular to EV miRs (miR -21, -29a, and -92a) by CRC stage varies by miR. For miR-21 and miR-29a, the non-vesicular serum levels did not show a particular trend with CRC stage, but there was a trend for decreasing levels of EV of these miRs which resulted in the increasing non-vesicular to EV ratios of these miRs by CRC stage. For miR-92a, the non-vesicular levels increased more than the EV levels by CRC stage which also led to increasing non-vesicular to EV ratios with CRC stage. These different relationships between non-vesicular and EV levels of miR -21, -29a, and -92a suggest distinct mechanisms of regulation of these miRs between these two compartments. These differences between non-vesicular and EV levels of miR-21 and miR-29a, and miR-92a by CRC stage argue against these results being artifactual. The receiver-operator analyses of these three miRs shows particular promise for miR-21 as the AUC was 0.76 in differentiating all stages of CRC versus controls and 0.73 in differentiating early CRC stages. Our study suggests that the sensitivity for detection of early stages of CRC may be increased by comparing specific CRC-related miR species in non-vesicular blood fractions and circulating extracellular vesicles. 

Most cancer studies on the overexpression of miR in the blood of cancer patients have studied their levels in exosomes, however one study did show that the majority of miR species studied in blood is non-exosomal (Turchinovich et al., 2011). Uratani et al., (2016) reported large differences in total serum and exosomal levels of miR-21, miR- 29a, miR-92a and miR-135b from patients with colorectal adenomas and colorectal adenocarcinomas, however, there was no information regarding the differences in non-vesicular and EV levels of miR-21, miR- 29a, miR-92a and miR-135b between various stages of CRC in their study. Extra-exosomal miR are highly stable in blood due to their relative resistance to RNAses, but especially so when complexed with Ago_2_, a protein involved in RNA silencing (Ng et al., 2009; Luo et al., 2013). Exosome release from cancer cells into the circulation is well documented (Ostenfeld et al., 2014) and exosomes contain the intracellular trafficking machinery to export macromolecules including miRs (Sohel et al., 2013; Urbanelli et al., 2013). For EV originating from cancer cells and circulating in blood, either their degradation or export of miRs could lead to the accumulation of miRs in blood. Although we are unable to identify the tumor as the source of the EV, serum miR levels may be useful for CRC screening regardless of their source. 

The strengths of our study are the validation of miR -21, -29a, and -92a for CRC detection in a previously untested ethnically diverse cohort, the focus on early stage CRC detection and the discovery of the strong relationship between the ratio of non-vesicular to EV miR -21, -29a, and -92a levels with CRC stage. The limitations of our study are the relatively small size and modest sensitivity of CRC detection. Our study shows that further studies in a larger population are needed to confirm the utility of the ratio of non-vesicular to EV levels of miRs for cancer screening. Future studies could look at the strength of combining various noninvasive (e.g. miR plasma SEPT9 methylation, fecal immunochemical hemoglobin) assays for the early detection of CRC or even advanced colorectal polyps, the precursors to CRC. Efforts along these lines will improve the acceptability of CRC screening.
